# Doxycycline pharmacokinetics in geese

**DOI:** 10.1111/jvp.13002

**Published:** 2021-07-27

**Authors:** Irene Sartini, Beata Łebkowska‐Wieruszewska, Andrzej Lisowski, Amnart Poapolathep, Andrejs Sitovs, Mario Giorgi

**Affiliations:** ^1^ Department of Veterinary Medicine University of Sassari Sassari Italy; ^2^ Department of Pharmacology Toxicology and Environmental Protection University of Life Sciences Lublin Poland; ^3^ Institute of Animal Breeding and Biodiversity Conservation University of Life Sciences Lublin Poland; ^4^ Department of Pharmacology Faculty of Veterinary Medicine Kasetsart University Bangkok Thailand; ^5^ Department of Pharmacology Riga Stradins University Riga Latvia; ^6^ Department of Veterinary Sciences University of Pisa Pisa Italy; ^7^ Department of Veterinary Medicine School of Veterinary Sciences University of Sassari Sassari Italy

**Keywords:** doxycycline, goose, *in silico*, pharmacokinetics

## Abstract

The study aims to describe the pharmacokinetics of doxycycline after a single intravenous and oral dose (20 mg/kg) in geese. In addition, two multiple‐dose simulations have been performed to investigate the predicted plasma concentration after either a 10 or 20 mg/kg daily administration repeated consecutively for 5 days. Ten geese were enrolled in a two‐phase cross‐over study with a washout period of two weeks. All animals were treated intravenously and orally with doxycycline, and blood samples were collected up to 48 h after drug administration. Sample analysis was performed using a validated HPLC‐UV method. A non‐compartmental approach was used to evaluate the pharmacokinetic parameters of the drug. A long elimination half‐life was observed (13 h). The area under the curve was statistically different between the two treatments, with the oral bioavailability being moderate (43%). The pharmacokinetic/pharmacodynamic index (%T>MIC) during the 48 h treatment period in the present study (71%) suggests that doxycycline appears to have therapeutic efficacy against some Mycoplasma species in the goose. The multiple‐dose simulations showed a low accumulation index. A dosage of 10 mg/kg/day for 5 days seemed to be adequate for a good therapeutic efficacy without reaching unnecessarily high plasma concentrations.

## 1 INTRODUCTION

Infection with Mycoplasma species is one of the main causes of significant economic loss in the domestic goose industry worldwide, causing reduced growth and egg production, and increased mortality and treatment costs (Stipkovits and Szathmary, 2012; Yadav et al., 2021). Waterfowl meat and eggs are highly valued in different countries worldwide, primarily due to their high nutritional quality. France, for instance, is the world's second‐largest duck and goose meat consumer, being exceeded only by China (Anonymous, 2021; Pingel, 2004). Moreover, wild birds, included ducks and geese, may play an important role as potential reservoirs and vectors of Mycoplasma species to the detriment of other domestic poultry. *M*. *gallisepticum* and *M*. *synovitis* are the most relevant strains among Mycoplasma species in poultry and are recognized as respiratory pathogens by the World Organization for Animal Health (OIE, 2018). Mycoplasma species are sensitive to a variety of antibacterials, including tetracyclines. Doxycycline is a semisynthetic tetracycline derivative with a broad spectrum of bacteriostatic activity. It acts by binding to the bacterial 30S ribosomal subunit, inhibiting protein synthesis in a time‐dependent manner (Nguyen et al., 2014). Doxycycline has a higher relative lipophilicity compared with older tetracyclines, resulting in advantages including high oral absorption and a wide volume of distribution (Saivin & Houin, 1988). It is active against *Chlamydia spp*., *Rickettsia spp*., *Mycoplasma spp*., *Pasteurella Multocida*, *Escherichia coli* and protozoa (Ismail & El‐Kattan, 2004; Pijpers et al., 1989; Yang et al., 2014). Doxycycline shows excellent effectiveness against *M*. *gallisepticum* strains *in vitro* (Zhang et al., 2016).

Doxycycline is indicated for the prevention and treatment of respiratory and gastrointestinal infections in poultry caused by different bacterial pathogens (EMA, 2010). Further, respiratory distress in geese is an indication for doxycycline, even though there are few and poorly documented studies to support this indication (EMA, 2010). Doxycycline is used in poultry at doses of 10–20 mg/kg for 3–5 days, provided as water‐soluble doxycycline hyclate powders or oral solutions for administration via drinking water (EMA, 2010).

The pharmacokinetics of doxycycline have been established in various avian species including chickens (Anadón et al., 1994; El‐Gendi et al., 2010; Laczay et al., 2001; Soliman et al., 2015; Yang et al., 2016, 2018), ostriches (Abu‐Basha et al., 2006), ducks (Bratoev et al., 2016; Yang et al., 2015) and turkeys (Santos et al., 1996), but to the best of the authors’ knowledge, no pharmacokinetic data on the goose is available.

The aim of this study was to determine the pharmacokinetics of doxycycline following a single intravenous (IV) and oral (PO) 20 mg/kg dose in the goose. In addition, two simulations of multiple‐dose treatments at 10 and 20 mg/kg administered daily for 5 days have been carried out to determine the predicted plasma concentrations.

## 2 MATERIALS AND METHODS

### 2.1 Chemicals and reagents

Doxycycline and oxytetracycline (internal standard, IS) powders with a standard purity of 99.0% were purchased from Sigma‐Aldrich. High‐performance liquid chromatography (HPLC)‐grade acetonitrile was purchased from Merck (Kenilworth). Trifluoracetic acid (TFA) was obtained from VWR International Bvba (Leuven, Belgium). Deionized water was produced using a Milli‐Q Millipore Water System (Millipore).

### 2.2 Animal treatment

Ten male Bilgoraska geese underwent a two‐phase cross‐over study design with a washout period of two weeks. The animals were approximately 2 years of age and their median body weight (BW) was 3.21 kg (2.88–4.28 kg).

All animals were judged to be in good health based on physical examination, serum chemistry and haematological analyses performed before the study commencement. The geese were monitored daily through observation of behaviour and appetite. They were acclimatised for 1 week in a 60 m^2^ enclosed area with an indoor shelter of 8 m^2^ before beginning the study. Animals could graze freely during the day as a ring with an identity code was applied to the left leg for easy identification. Geese were fed with a drug‐free pelleted diet twice a day and water was supplied *ad libitum*.

Geese were randomly divided in two groups. In the first phase, group 1 (*n *= 5) was treated with 20 mg/kg doxycycline (Doxycyclinum TZF (0.02 g/ml), Polfa SA Tarchomin, Warszawa, Poland) IV using a sterile 20‐gauge 3.75 cm needle in the left ulnar vein, while group 2 (*n *= 5) received a single oral dose of doxycycline (20 mg/kg) (Doxycyclinum 200 Biofaktor, 0.2 g/g, Biofaktor, Skierniewice, Poland) by crop‐gavage. The powder was dissolved in sterile water at a concentration of 40 g/l for an easily administration. In the second phase, the groups were inverted, with group 2 receiving doxycycline IV and group 1 receiving doxycycline PO at the same dosages.

Blood samples (approximately 1 ml) were collected from a pre‐implanted 22‐gauge catheter in the right ulnar vein. After each sample collection, the catheter was flushed with 1 ml of 0.9% saline containing 10 IU/ml heparin. Prior to each blood collection, the first 0.2 ml of blood was discarded. Blood was collected at 0 (before drug treatment), 0.085, 0.25, 0.5, 0.75, 1, 1.5, 2, 4, 6, 8, 10, 24 and 48 h after IV administration. After PO administration, blood was collected at 0 (before drug treatment), 0.025, 0.5, 0.75, 1, 1.5, 2, 4, 6, 8, 10, 24 and 48 h. Blood was collected in heparinized tubes and centrifuged at 1500 *g*. The harvested plasma was stored at −20°C and analysed within 30 days of collection.

### 2.3 Sample preparation

Sample purification was performed using protein precipitation. 200 µl of plasma was spiked with 20 µl of IS (10 µg/ml) solution in water. After the addition of 1 ml of acetonitrile and 20 µl of TFA, each sample was vortexed, shaken at 60 oscillations/min for 10 min and centrifuged at 4000 *g* for 10 min. 1 ml of the upper layer was transferred into a clean tube and dried at 45 °C under a gentle nitrogen stream. The residue was dissolved in 200 µl of mobile phase and vortexed, and an aliquot of 50 μl was injected onto the HPLC system.

### 2.4 HPLC conditions

The HPLC system was a LC Jasco consisting of a ternary gradient system (PU 980), in line degasser (DG‐2080‐53), autosampler (AS‐2055) and an UV multiple wavelength detector (MD‐1510). The chromatographic separation assay was performed with a Luna C18 analytical column (250 × 4.6 mm inner diameter, 5 μm particle size, Phenomenex) maintained at 30 °C using a Peltier system (CO‐4062) (Jasco). The mobile phase consisted of acetonitrile:0.1% TFA (21:79% v:v) in water with a flow rate of 1 ml/minutes. The optimal wavelength for the quantification was set at 350 nm.

### 2.5 Validation of the analytical method

The quantitative HPLC method was fully validated for goose plasma in terms of linearity, intra‐day and inter‐day precision, recovery, limits of detection (LOD) and lower limit of quantification (LLOQ), according to the EMA guidelines (Anonymous, 2012). Doxycycline (1 mg/ml) and IS (1 mg/ml) stock solutions and all related dilutions were produced in water. Linearity was assessed using goose plasma spiked with low (0.1, 0.25, 0.5, 1, 5, 10 µg/ml) or high (10, 25, 50, 100, 250, 500 µg/ml) concentrations. Three replicates of each concentration were analysed, with two calibration curves constructed using standard doxycycline concentrations *vs*. ratio of doxycycline/IS peak areas. Intra‐day and inter‐day precision were calculated after analysis of six plasma samples spiked with doxycycline at three different concentrations (QC; 0.25, 10, and 250 μg/ml) and expressed as the percentage coefficients of variation (CV, %). Sample recovery was evaluated by comparing the response (in area) of high (250 µg/ml), middle (10 µg/ml), low (0.25 µg/ml) concentration spiked samples and the IS to the response of equivalent standards. Recovery is expressed as mean ± standard deviation (SD). The LOD was estimated as the plasma drug concentration that produced a signal‐to‐noise ratio of three and LLOQ was determined as the lowest plasma concentration that produced a signal‐to‐noise ratio of five. The mean concentration was within 15% and 20% of the nominal values for the QCs and LLOQ samples, respectively.

### 2.6 Pharmacokinetics and statistical analysis

The data were pharmacokinetically analysed using a non‐compartmental approach (ThothPro™^T^ 4.3; ThothPro LLC, Poland).

The maximum plasma concentration (C_max_) and time to reach it (T_max_) were determined directly from the concentration *vs*. time curves. The elimination half‐life (t_1/2_) was calculated using least squares regression analysis of the concentration‐time curve. The area under the curve (AUC) was calculated by linear log trapezoidal (IV administration) and the linear‐up log‐down rule (PO administration). From these values, the volume of distribution at steady state (Vss =dose x AUMC/AUC^2^) and clearance (Cl =dose/AUC) were calculated. The individual value of AUC_rest%_ was lower than 20% of AUC_(0‐∞),_ and the square of coefficient of determination (R^2^) of the terminal phase regression line was >0.85.

The absolute oral bioavailability (F) was calculated as:

$$F\left(\%\right)=\frac{AUC_{\mathrm{PO}}}{AUC_{\mathrm{IV}}}\times 100$$

The extraction ratio (E) for doxycycline after IV administration was calculated for each goose as clearance divided by cardiac output (Grubb, 1983; Toutain and Bousquet‐Mélou, 2004a), where cardiac output (ml/min) was calculated as body weight (kg) to the power of 0.69 multiplied by 290.7 (Grubb, 1983).

The modelling of a daily oral dose regimen of 10 and 20 mg/kg/day administered for 5 days was computed applying the superposition principle and assuming first‐order kinetics (Gabrielsson & Daniel, 2016) using ThothPro™ software (ThothPro™ 4.3; ThothPro LLC, Poland).

The potential accumulation ratio (R) at 24 h dosing intervals (τ) following both simulations was determined using the following formula (Toutain & Bousquet‐Mélou, 2004b):

$$R=\frac{1}{\left[1‐{\left(0.5\right)}^{\tau /t_{1/2}}\right]}$$

Fluctuations of drug plasma concentration at the steady‐state peak and trough concentrations were calculated with the following equation:

$$\frac{P}{T}=\frac{C_{\max_{\mathrm{ss}}}}{C_{\min_{\mathrm{ss}}}}=2^{\tau /t_{1/2}}$$

where P/T is the peak/trough concentration ratio at steady state and Cmax_ss_ and Cmin_ss_ are the steady‐state peak and trough concentrations, respectively (Toutain and Bousquet‐Mélou, 2004b).

The pharmacokinetic parameters are reported as geometric mean and ranges, except for T_max_ (categorical variable) which is expressed as the median value and range (Julious & Debarnot, 2000).

Wilcoxon's rank‐sum test was used to statistically compare the pharmacokinetic data between the two routes of administration (Powers, 1990).

## 3 RESULTS

### 3.1 Validation of the analytical method

The analytical method demonstrated linearity in the low and high concentration ranges, with R^2^ of 0.997 (y = 0.7868x–0.1987) and 0.999 (y = 0.9913x–0.5293), respectively. The LOD and LLOQ were 0.03 and 0.1 µg/ml, respectively, and the mean extraction recovery was 96% ± 17%. The inter‐ and intra‐day precision showed a CV% of 15.3 and 9.1, respectively. For the LLOQ, the CV% was lower than 20%.

### 3.2 Pharmacokinetic results

No adverse effects were observed during or after drug administration in any of the geese.

**FIGURE 1:**
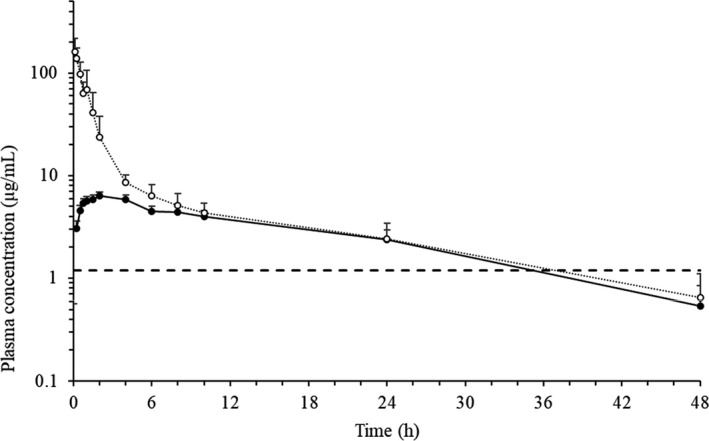
Semi logarithmic doxycycline plasma concentration‐time curves after a single 20 mg/kg IV (*n *= 7, ⋅⋅⋅○⋅⋅⋅) or PO (*n *= 10, —●—) dose in geese. The horizontal dashed line (– – –) represents the MIC value for *M*. *gallisepticum* (1.20 μg/ml)

Plasma doxycycline concentrations were always higher than the LLOQ of the analytical method (Figure 1). The elimination slope of the IV and PO plasma concentration‐time curves was similar, with a long elimination t_1/2_ (13.95 h, IV and 13.35 h, PO) (Table 1). Three animals showed an AUC_rest%_ higher than 20% in the IV treatment, and so were excluded from the IV pharmacokinetic assessment. Cl was slow (0.07 ml/g h), and the V_ss_ was moderate (0.58 ml/g). The oral F was moderate (42.79%), with a significant difference between AUC_IV_ and AUC_PO_. The extraction ratio was low (2%).

**TABLE 1:** Pharmacokinetic parameters of doxycycline after a single 20 mg/kg IV (*n *= 7) or PO (*n *= 10) dose in geese

		IV			PO		
		Geometric mean	Min	Max	Geometric mean	Min	Max
AUC_(0‐t)_	µg h/ml	273.99	202.70	411.05	120.1*	73.26	173.07
AUC_(0‐inf)_	µg h/ml	287.62	214.20	427.24	131.2*	82.98	187.48
k_el_	1/h	0.05	0.04	0.06	0.05	0.05	0.06
t_1/2_	h	13.95	11.73	17.84	13.35	11.98	15.13
C_max_	μg/ml	/	/	/	6.67	4.45	8.99
T_max_	h	/	/	/	2.00	1.00	4.00
Cl	ml/g h	0.07	0.05	0.10	/	/	/
V_ss_	ml/g	0.58	0.31	1.03	/	/	/
F	%	/	/	/	42.79	31.62	54.14

Note

AUC_(0‐t)_, area under the curve from zero to the last detectable timepoint; AUC_(0‐inf)_, area under the curve from zero to infinity; k_el_, elimination rate constant; t_1/2_, terminal half‐life; C_max_, maximum concentration; T_max_, time at maximum plasma concentration; V_ss_, volume of distribution; Cl, plasma clearance; F, bioavailability./= Not applicable. *Significantly different between the groups *(p *< .05).

**FIGURE 2:**
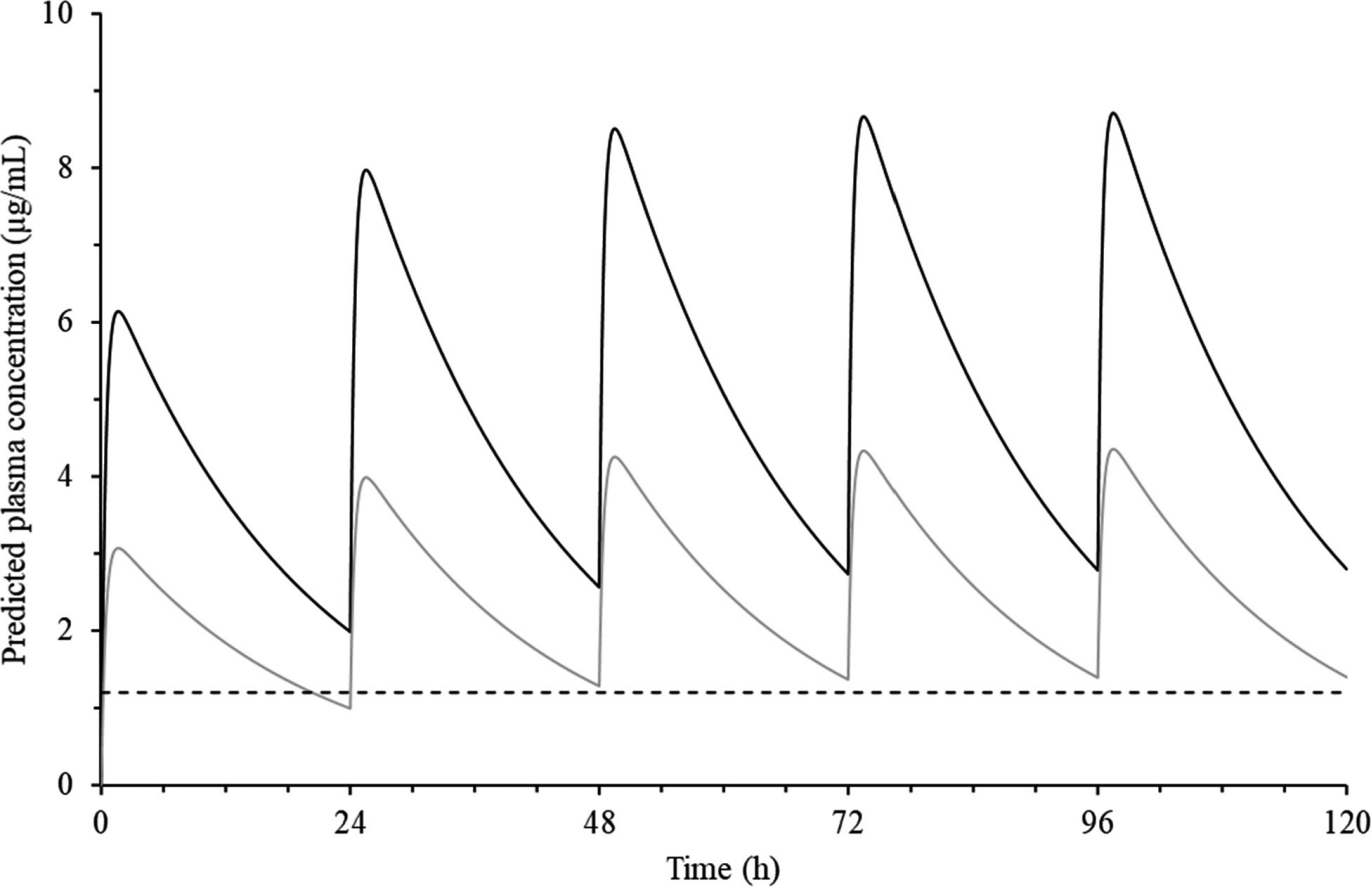
Multip‐e dose simulation of doxycycline PO administered to geese at 10 mg/kg (grey line, 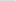) and 20 mg/kg (black line, **—**) daily for 5 days. The horizontal dashed line (**– – –**) represents the MIC value for *M*. *gallisepticum* (1.20 μg/ml)

Figure 2 shows the predicted doxycycline plasma concentration vs. time curves, obtained from the multiple‐dose simulation of 10 and 20 mg/kg/day repeated for 5 days. After the third dose, the steady state was reached with a P/T value of 3.43. The accumulation index was 1.4.

## 4 DISCUSSION

To the best of the authors’ knowledge, this is the first study which reports the pharmacokinetics of doxycycline in geese.

The pharmacokinetic profile of doxycycline in geese was similar to that reported in ducks by Yang et al. (2015). The Cl was similar (ducks, 0.06 ml/g h; geese, 0.07 ml/g h); however, the half‐life was longer (ducks, 21 h; geese, 13 h). The half‐life in geese was similar to that found in broiler chickens (13.9 h, Soliman et al., 2015; 14.9 h, Hsiao et al., 2016; 13.9 h, Hantash et al., 2008). The V_ss_ in geese (0.58 ml/g) was comparable to most other avian species (ducks, 0.59 mL/g, Yang et al., 2015; laying hens, 0.87 ml/g, Yang et al., 2016), however, higher than reported in chickens by Anadón et al. (1994) (0.11 ml/g). It has been demonstrated that waterfowl have physiological differences in renal morphology compared to galliform birds, which may result in species differences in renal elimination and/or reabsorption of drugs (Warui, 1989). Differences in the activity of liver enzymes between waterfowl and galliform birds may also be a potential cause in the difference in the V_ss_ (Warui, 1989).

The absorption profile does not differ appreciably from that observed in chickens given the same dose (Hantash et al., 2008; Hsiao et al., 2016; Soliman et al., 2015). In chickens, the peak plasma concentrations in three studies (4.7 µg/ml, 4.5 µg/ml, 5.4 µg/ml) occurred at 1.30 h, 2.07 h and 3.60 h, respectively (Hantash et al., 2008; Hsiao et al., 2016; Soliman et al., 2015), whereas in geese the C_max_ (6.67 µg/ml) was reached in 2 h. Substantial differences were observed with the results of Anadón et al., 1994, where the C_max_ in broiler chickens (also administered 20 mg/kg) was much higher than that reported in geese (54 µg/ml) and achieved much faster (0.35 h). Even ducks, another waterfowl species, had a different C_max_ (17.57 µg/ml) when treated at the same dosage (Yang et al., 2015). Discrepancies in the experimental methodology, including the different drug formulation used (different excipients) may have contributed to these differences (Toutain & Bousquet‐Mélou, 2004c). For example, the present study administered an oral powder formulation for poultry to geese, whereas a commercial doxycycline hyclate formulation for injection was used orally in ducks (Yang et al., 2015). Additionally, differences in the characteristics of the animals such as the age (geese, 2 years; ducks, 6 months) or body weight (geese, 3.31 kg; ducks, 1.52 kg), or differences in the status of the animals (e.g. feeding conditions, diet) may have also influenced the drug absorption (Yang et al., 2015).

Oral bioavailability of doxycycline in different avian species has consistently been reported as moderate, both in the current study (43%) and in the literature: 39% in the duck (Yang et al., 2015), 52% in the laying hen (Yang et al., 2016) and 41% in the broiler chicken (Anadón et al., 1994).

The pharmacokinetic/pharmacodynamic (PK/PD) index T>MIC (the duration of plasma concentrations exceeding the MIC) has been proposed to predict the success of doxycycline therapy as it is a time‐dependent antibiotic (Toutain et al., 2002). MIC values reported in the literature for doxycycline against different Mycoplasma species isolated from geese and ducks vary significantly between strains (Grózner et al., 2016; Gyuranecz et al., 2020). *Mycoplasma gallisepticum* and *Mycoplasma synoviae* are considered the most relevant pathogens in the poultry industry, with reported MIC values in avian species of 1.20 µg/ml (Zhang et al., 2017) and 0.625–1 µg/ml (Catania et al., 2019; Kreizinger et al., 2017), respectively. The optimal %T>MIC value has been reported as 54.36% during a 48 h treatment period with doxycycline (Zhang et al., 2016). In the present study, doxycycline plasma concentration remained above the MIC value of 1.2 µg/ml almost 34 h after PO administration, exceeding the PK/PD index (71%).

Since a multiple‐dose schedule in the range of 10–20 mg/kg is used in practical clinical conditions, two simulations were carried out to predict the plasma concentrations reached after 5 day's treatment with these doses (10–20 mg/kg/day). The multiple‐dose simulation showed that steady state was reached after the third dose. An accumulation index of 1.42 was found, suggesting a slight plasma accumulation. The predicted plasma concentration after both simulations exceeded the MIC (1.2 μg/ml) value (Zhang et al., 2017), suggesting that doxycycline could be a promising therapeutic treatment in geese for Mycoplasma species. A dosage of 10 mg/kg/day for 5 days seems to be adequate to reach the appropriate plasma levels for clinical efficacy without the need for higher doses and unnecessarily high plasma concentrations (20 mg/kg/day simulation).

Further consideration should be made of the following: (1) since in practice doxycycline would typically be given in drinking water, the drug/water intake could differ between animals. It is therefore reasonable to hypothesize that in a practical context the predicted plasma concentration may be lower than that found in the present simulation, with a lower P/T ratio (Sartini et al., 2020, 2021). Further, the duration of medicated water availability could affect T>MIC and clinical efficacy; (2) the protein plasma binding of doxycycline in geese is not available in the literature and was not evaluated in the present study. In many species, it is reported to be high, so may be an important factor in PK/PD analysis; (3) the presence of resistant strains and cross‐resistance phenomena may result in ineffective drug treatment in cases where a higher MIC is required. Some studies reported Mycoplasma strains requiring an MIC value >10 µg/ml for doxycycline (Grózner et al., 2016; Kreizinger et al., 2017). Thus, it is fundamental to highlight the importance of susceptibility testing before therapy commencement and antimicrobial stewardship.

## 5 CONCLUSION

Doxycycline showed a long half‐life with a moderate bioavailability after oral administration. The PK/PD index in the 48 h after a single PO treatment of 20 mg/kg doxycycline (%T>MIC 71%) suggests this dose would be effective against some Mycoplasma species in the goose. However, further studies are needed to clarify the free fraction of the drug. The multiple‐dose simulations aimed to reflect clinical use in poultry, and these showed a low accumulation index. A dosage of 10 mg/kg/day for 5 days seems to be adequate for good therapeutic efficacy without achieving unnecessarily high plasma concentrations. Due to the potential variability in drug intake associated with drinking water dosing in clinical practice, and the possible presence of resistant pathogen species, further studies are warranted to confirm these findings.

## CONFLICT OF INTEREST

None of the authors has any financial or personal relationships that could inappropriately influence or bias the content of this paper.

## AUTHORS’ CONTRIBUTIONS

I.S. carried out the sample preparation and analyses, contributed to the animal experiment, to the pharmacokinetic analysis and writing the manuscript. B.LW. carried out the animal study and writing of the manuscript. A.L. contributed to the animal experiment. A.P. contributed to the data analysis and interpretation. A.S. contributed to the sample preparation and analyses. M.G. conceived the presented idea, directed the study, contributed to the animal experiment, interpretation of the results and writing of the manuscript. All authors have read and approved the final manuscript.

## ANIMAL WELFARE AND ETHICS STATEMENT

The authors confirm that the ethical policies of the journal were followed. The Institutional Animal Care and Use Committee of the University of Lublin (Poland) approved the study protocol according to the provision of the EC council Directive 2010/63.

## Data Availability

The data concerning the findings of this study are available from the corresponding author upon request.
